# CT features based preoperative predictors of aggressive pathology for clinical T1 solid renal cell carcinoma and the development of nomogram model

**DOI:** 10.1186/s12885-024-11870-1

**Published:** 2024-01-30

**Authors:** Keruo Wang, Liang Dong, Songyang Li, Yaru Liu, Yuanjie Niu, Gang Li

**Affiliations:** 1 Department of Urology, Tianjin Institute of Urology, The Second Hospital of Tianjin Medical University, Tianjin, China; 2 Department of Pulmonary & Critical Care Medicine, 8th Medical Center, Chinese PLA General Hospital, Beijing, China

**Keywords:** Carcinoma, renal cell, Computed tomography (CT), Pathology, Surgical, Nomogram

## Abstract

**Background:**

We aimed to identify preoperative predictors of aggressive pathology for cT1 solid renal cell carcinoma (RCC) by combining clinical features with qualitative and quantitative CT parameters, and developed a nomogram model.

**Methods:**

We conducted a retrospective study of 776 cT1 solid RCC patients treated with partial nephrectomy (PN) or radical nephrectomy (RN) between 2018 and 2022. All patients underwent four-phase contrast-enhanced CT scans and the CT parameters were obtained by two experienced radiologists using region of interest (ROI). Aggressive pathology was defined as patients with nuclear grade III-IV; upstage to pT3a; type II papillary renal cell carcinoma (pRCC), collecting duct or renal medullary carcinoma, unclassified RCC or sarcomatoid/rhabdoid features. Univariate and multivariate logistic analyses were used to determine significant predictors and develop the nomogram model. To evaluate the accuracy and clinical utility of the nomogram model, we used the receiver operating characteristic (ROC) curve, calibration plot, decision curve analysis (DCA), risk stratification, and subgroup analysis.

**Results:**

Of the 776 cT1 solid RCC patients, 250 (32.2%) had aggressive pathological features. The interclass correlation coefficient (ICC) of CT parameters accessed by two reviewers ranged from 0.758 to 0.982. Logistic regression analyses showed that neutrophil-to-lymphocyte ratio (NLR), distance to the collecting system, CT necrosis, tumor margin irregularity, peritumoral neovascularity, and RER-NP were independent predictive factors associated with aggressive pathology. We built the nomogram model using these significant variables, which had an area under the curve (AUC) of 0.854 in the ROC curve.

**Conclusions:**

Our research demonstrated that preoperative four-phase contrast-enhanced CT was critical for predicting aggressive pathology in cT1 solid RCC, and the constructed nomogram was useful in guiding patient treatment and postoperative follow-up.

## Introduction

The use of cross-sectional imaging techniques has significantly increased the likelihood of incidental detection of cT1 renal tumors. In most cases, surgery is required to treat T1 RCC. PN is the preferred treatment measure because it can better preserve kidney function and provide similar oncologic outcomes compared to RN [1]. Although PN offers the chance of cure for T1 RCC patients, it is crucial to elucidate the impact of pathological aggressiveness on patient prognosis based on postoperative histological findings [2]. The aggressive pathological features, including nuclear grade III/IV, pT3a upstage, sarcomatoid dedifferentiation, and aggressive pathological subtypes are associated with advanced disease and poorer outcomes [3, 4]. Apart from surgical treatment, active surveillance and tumor ablation have become important options for the management of T1 RCC patients [5]. Existing literature suggests that T1 RCC patients with complications or indolent pathology have low cancer-specific mortality. In such cases, active surgical treatment may not improve overall survival (OS) or cancer-specific survival (CSS) compared to active surveillance or tumor ablation [6–8]. As a consequence, the prediction of aggressive pathology for T1 RCC is essential to determine the patient’s treatment plan and follow-up schedule.

In general, most renal masses can be detected and characterized by imaging such as ultrasound, CT, and MRI. Abdominal contrast-enhanced CT scan is the most common imaging method used to evaluate renal tumors and is known for its predictive value in determining nuclear grade, pT3a upstage, and pathological subtypes of RCC [9–11]. Renal mass biopsy (RMB) has been performed in recent years to reveal histological characteristics of radiologically indeterminate renal masses. However, the accuracy and safety of RMB are of concern due to the possibility of intratumoral heterogeneity and biopsy tract seeding [12, 13].

On four-phase contrast-enhanced CT, distinguishing between benign renal tumors and RCC is relatively straightforward based on features such as macroscopic fat, enhancement characteristics, necrosis, and others [14]. Most relevant studies have focused on differentiating specific subtypes of benign renal tumors, like fat-poor angiomyolipoma/oncocytomas, from RCC [14]. The choice of treatment for T1 RCC patients depends on the degree of pathological aggressiveness and the Bosniak grading system in determining the pathological malignancy and aggressiveness of cystic renal masses is accurate. Consequently, our study aimed at predicting the aggressive pathology of cT1 solid RCC and retrospectively enrolled cases with pathologically confirmed diagnoses of RCC. We analyzed the imaging characteristics of 776 cT1 solid RCC patients, including qualitative information such as tumor margin regularity, CT necrosis, calcification, etc., as well as quantitative parameters such as attenuation values of different ROI, and constructed a nomogram model.

## Materials and methods

### Patients

**Fig. 1:**
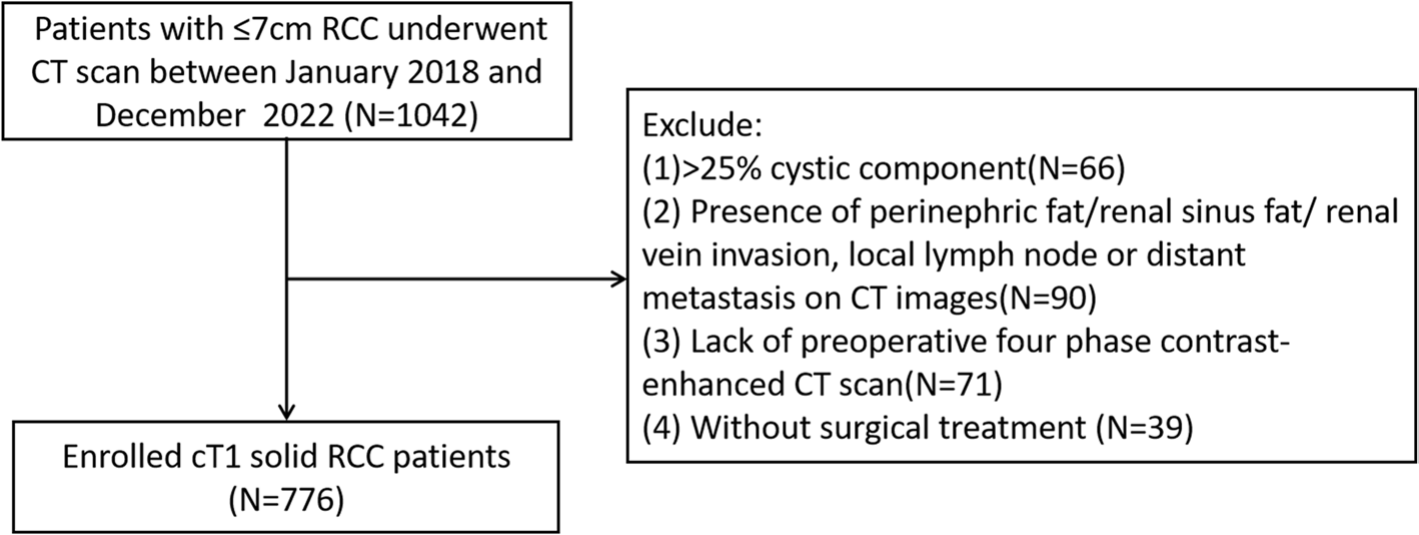
Flow chart of patient inclusion and exclusion criteria

Our retrospective study consecutively enrolled 776 patients diagnosed with cT1 RCC treated in the Second Hospital of Tianjin Medical University between January 2018 and December 2022. To be eligible for our study, patients should meet the following inclusion criteria: (1) Renal mass ≤ 7 cm in maximum diameter; (2) Postoperative pathology confirmed RCC; (3) The patients who underwent contrast-enhanced CT scan, including pre-contrast phase (PCP), corticomedullary phase (CMP), nephrographic phase (NP), and excretory phase (EP) in our medical center. The exclusion criteria were as follows: (1) Renal mass with > 25% cystic component; (2) Presence of perinephric fat/sinus fat/renal vein invasion, local lymph node, or distant metastasis on CT images; (3) Lack of preoperative four-phase contrast-enhanced CT scan; (4) Without surgical treatment (Fig. 1).

### Clinicopathological diagnosis

We collected the clinical factors of cT1 solid RCC patients, including age, gender, laterality, body mass index (BMI), ECOG performance status, symptomatic presentation, hypertension, diabetes, and smoking history. We also collected laboratory test results and surgical findings, including hemoglobin, NLR, surgical approach, and type of nephrectomy. Pathological findings included clinical tumor size, cT stage (AJCC 8th, 2017), histology subtype, surgical margin status, tumor nuclear grade (WHO/ISUP, 2022), pT3a upstage, and sarcomatoid/rhabdoid component. The aggressive pathology of cT1 solid RCC was defined as follows: (1) nuclear grade III-IV; (2) upstage to pT3a; (3) non-clear cell subtypes with adverse prognosis (type II pRCC, collecting duct or renal medullary carcinoma, unclassified RCC); (4) with sarcomatoid/rhabdoid features [15–17].

### CT imaging evaluation

A GE Discovery 750 HD CT scanner was used to conduct renal contrast-enhanced CT from the top of the diaphragm to the anterior superior iliac spine. The CT scanning mode was used with the following parameters: the tube voltage was 120 kV, the tube current was 100 mA, and the reconstruction thickness and scanning thickness were both 1.25 mm. All patients performed PCP of the CT scan before CMP (25 ~ 30 s delay), NP (60 ~ 90 s delay), and EP (120 ~ 180 s delay). For the enhanced examination, 100 ml of the non-ionic contrast iodixitol (containing iodine 300 mg/ml) was injected through the cubital vein with an injection flow rate of 2.5 ml/s.

**Fig. 2:**
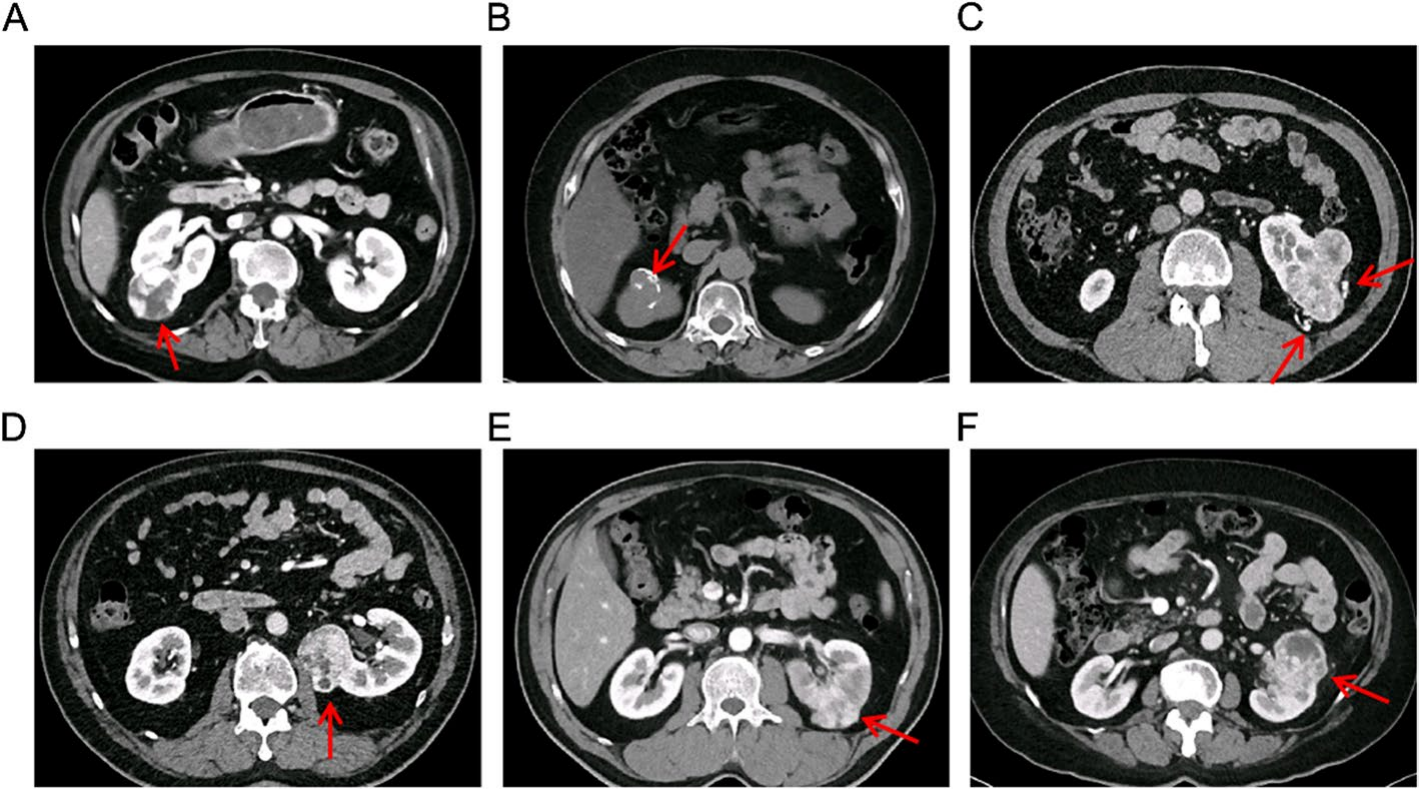
Representative images on CT for cT1 solid RCC: (**A**) necrosis, (**B**) calcification, (**C**) peritumoral neovascularization, (**D**) tumor margin irregularity: nodular growth pattern, (**E**) tumor margin irregularity: blurred boundary between renal tumor and parenchyma, (F)tumor margin irregularity: completely non-elliptical shape.  RCC, renal cell carcinoma

All CT images were analyzed at a picture archiving and communication system workstation (PACs) and assessed by two radiologists with 6 and 10 years of experience in urological imaging blinded to the pathological results. The qualitative CT features of RCC are as follows: maximal tumor diameter (≤ 4 cm/4 cm ~ 7 cm), exophytic/endophytic rate (≥ 50%/< 50%/Endophytic), distance to the collecting system (> 7 mm/4 mm ~ 7 mm/≤ 4 mm), polar location (entirely above or below the polar line/cross the polar line/> 50% cross the polar line, cross the axial renal midline or entirely between the polar lines) [11], necrosis [18], calcification [19], tumor margin regularity [10] and peritumoral neovascularity [20]. The examples of intratumoral necrosis, calcification, tumor margin irregularity, and peritumoral neovascularity are shown in Fig. 2.

**Fig. 3:**
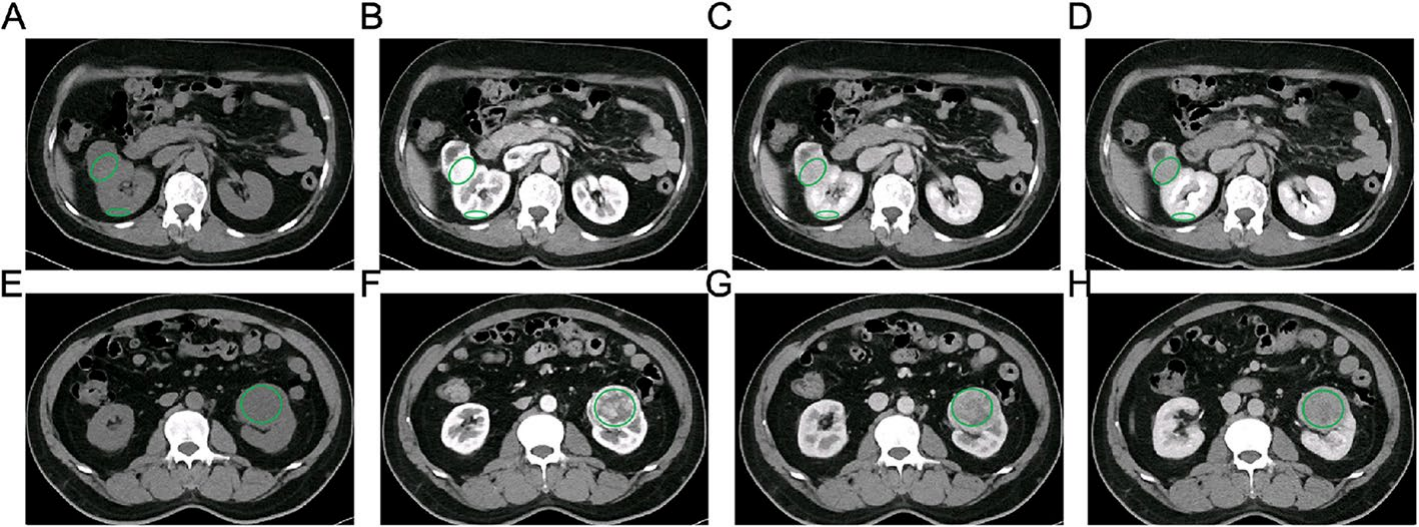
The placement method of ROIs for measuring TAV and TAC: (**A**) PCP, (**B**) CMP, (**C**) NP, (**D**) EP, and for measuring HDT: (**E**) PCP, (**F**) CMP, (**G**) NP, (**H**) EP.  ROI: region of interest; PCP: precontrast phase; CMP: corticomedullary phase; NP: nephrographic phase; EP: excretory phase

The attenuation values [Hounsfeld units (HU)] of ROIs in four-phase contrast-enhanced CT were also recorded, which represented the radiodensity of tissues. The attenuation values of renal tumor (TAV) and renal cortex (TAC) were measured on the same axial image of contrast-enhanced CT. TAV_PCP/CMP/NP/EP_ and TAC_PCP/CMP/NP/EP_ represent the CT attenuation values of the renal tumor and adjacent renal cortex in PCP, CMP, NP, and EP, respectively. The final TAV and TAC are the averages measured by two radiologists. To eliminate the individual differences in CT images resulting from the metabolism of contrast agents, we additionally calculated the net enhancement value of renal tumor (TEV), the net enhancement value of renal cortex (CEV), and the relative enhancement ratio of renal tumor (RER). The TEV and CEV are calculated as follows: TEV_CMP/NP/EP_=TAV_CMP/NP/EP_-TAV_PCP,_ CEV_CMP/NP/EP_=TAC_CMP/NP/EP_-TAC_PCP_. The RER is calculated as follows: RER_CMP/NP/EP_=TAV_CMP/NP/EP_/TAC_CMP/NP/EP_. Two radiologists selected ROIs based on the following principles: (1) The ROIs of both the renal tumor and normal renal cortex were consistent in size and location on four-phase contrast-enhanced CT images. (2) The circular or elliptical ROI should include the relatively homogeneous and maximum enhancing solid region of RCC while avoiding intratumoral necrosis, calcification, vasculature, and cystic component. (3) Each area should be measured twice, and if there is more than one solid enhancement region, they should be measured separately and take the average eventually. An example of selecting the ROI for measuring TAV and TAC is shown in Fig. 3A-D. Furthermore, the heterogeneous degree of tumor (HDT) was determined based on the standard deviation (SD) of CT values. The selection criteria of ROI for measuring HDT are as follows (Fig. 3E-H): (1) Since the margin of the tumor was most clearly in NP, it was first measured in NP. The ROIs of RCC in PCP, CMP, and EP should be placed refer to NP. (2) The circular or elliptical ROIs should cover the full RCC regions as much as possible, with the margins 2–3 mm medial to the tumor border. (3) Each tumor image should be measured twice, with the average values being recorded.

### Statistical analysis

Our research provided information on the qualitative and quantitative data, with frequency (percentage) and median [interquartile range (IQR)] used respectively. The ICC score was used to assess agreement between two radiologists, with a score > 0.75 regarded as good reproducibility. The comparisons between cT1 solid RCC patients with aggressive and non-aggressive pathology were used by the Student t-test or Mann–Whitney U test for the continuous variables, and the Chi-square test or Fisher’s exact test for the categorical variables. Logistic regressions were used to identify the independent predictors of aggressive pathology. P values in univariate logistic regression were adjusted by the Benjamini-Hochberg FDR method, and the variables with adjusted p values less than 0.05 were considered statistically significant. Variation Inflation Factors (VIF, < 5 being considered non-significant) were performed to evaluate the collinearity of combinations of variables. To evaluate the discrimination performance of the nomogram, a ROC curve was used, while the AUC, calibration plots, and DCA were calculated to assess the accuracy, goodness of fit, and clinical benefit. The risk score and linear predicted probability of individual aggressive pathology were calculated using the significant risk factors and their corresponding regression coefficients. Subsequently, the individual predicted probability was used for risk stratification and subgroup analysis. All statistical analyses were performed using SPSS 22.0 and R software (version 4.3.1), and *p* < 0.05 was considered statistically significant.

## Results

### Clinicopathological and radiologic features

The study analyzed 776 patients with cT1 solid RCC who underwent PN/RN. Out of these, 250 patients had aggressive pathology. The clinicopathological characteristics of all patients are summarized in Table 1. The median age of the patients was 62.0 years and 72% were male. Among the 250 cT1 solid RCC patients with aggressive pathology, 210 (27.1%) had high nuclear grade, 58 (7.5%) had pT3a upstage, 25 (3.2%) had type II pRCC, collecting duct carcinoma, renal medullary carcinoma or unclassified RCC, and 4 (0.5%) had sarcomatoid/rhabdoid features. There were significant differences between the two groups in terms of age (*P* < 0.001), ECOG performance status (*P* = 0.005), hemoglobin (*P* < 0.001), NLR (*P* < 0.001), type of nephrectomy (*P* < 0.001), clinical tumor size (*P* < 0.001) and histology subtype (*P* < 0.001).

**Table 1:** Clinicopathological characteristics of enrolled patients

Variables	Overall (*N* = 776)	Non-aggressive pathology (*N* = 526)	Aggressive pathology (*N* = 250)	P
**Clinical findings**				
Age at surgery, year (IQR)	62.0 (53.0–69.0)	61.0 (52.0–68.0)	63.0 (56.0–71.0)	< 0.001
Gender: male (%)	559.0 (72.0)	377.0 (71.7)	182.0 (72.8)	0.744
Laterality: left (%)	388.0 (50.0)	267.0 (50.8)	121.0 (48.4)	0.539
ECOG performance status (%)				0.005
0–1	752.0 (96.9)	516.0 (98.1)	236.0 (94.4)	
2–4	24.0 (3.1)	10.0 (1.9)	14.0 (5.6)	
Symptomatic presentation (%)	246.0 (31.7)	155.0 (29.5)	91.0 (36.4)	0.052
Hypertension (%)	389.0 (50.1)	263.0 (50.0)	126.0 (50.4)	0.917
Diabetes (%)	170.0 (21.9)	109.0 (20.7)	61.0 (24.4)	0.247
Smoking history (%)	296.0 (38.1)	194.0 (36.9)	102.0 (40.8)	0.294
BMI, kg/m^2^ (IQR)	25.5 (23.5–27.8)	25.6 (23.7–27.8)	25.2 (23.1–27.6)	0.189
Hemoglobin, g/L (IQR)	138.0 (126.0-149.0)	139.0 (128.0-151.0)	134.0 (120.8–148.0)	< 0.001
NLR (IQR)	2.3 (1.6–4.4)	2.2 (1.6–3.5)	2.9 (1.9–7.3)	< 0.001
**Surgical findings**				
Surgical approach (%)				0.168
Open	17.0 (2.2)	15.0 (2.9)	2.0 (0.8)	
Laparoscopic	645.0 (83.1)	432.0 (82.1)	213.0 (85.2)	
Robotic	114.0 (14.7)	79.0 (15.0)	35.0 (14.0)	
Type of nephrectomy (%)				< 0.001
Radical nephrectomy	194.0 (25.0)	93.0 (17.7)	101.0 (40.4)	
Partial nephrectomy	582.0 (75.0)	433.0 (82.3)	149.0 (59.6)	
**Pathologic findings**				
Clinical tumor size, cm (IQR)	4.0 (3.1–5.1)	3.8 (2.8–4.9)	4.5 (3.5–5.5)	< 0.001
CT stage (%)				0.472
T1a	389.0 (50.1)	293.0 (55.7)	96.0 (38.4)	
T1b	387.0 (49.9)	233.0 (44.3)	154.0 (61.6)	
Histology subtype (%)				< 0.001
Clear cell RCC	705.0 (90.9)	490.0 (93.2)	215.0 (86.0)	
Papillary RCC	33.0 (4.3)	11.0 (2.1)	22.0 (8.8)	
Chromphobe RCC	24.0 (3.1)	19.0 (3.6)	5.0 (2.0)	
Others	14.0 (1.8)	6.0 (1.1)	8.0 (3.2)	
Surgical margin: positive (%)	12.0 (1.5)	9.0 (1.7)	3.0 (1.2)	0.590
Aggressive pathology (%)				
Tumor grade: III-IV	210.0 (27.1)	0.0 (0.0)	210.0 (84.0)	
PT3a upstage	58.0 (7.5)	0.0 (0.0)	58.0 (23.2)	
type II pRCC, collecting duct, renal medullary carcinoma, unclassified RCC	25.0 (3.2)	0.0 (0.0)	25.0 (10.0)	
Sarcomatoid/rhabdoid component	4.0 (0.5)	0.0 (0.0)	4.0 (1.6)	

*BMI *Body mass index, *NLR *Neutrophil-to-lymphocyte ratio, *RCC *Renal cell carcinoma, *pRCC *Papillary renal cell carcinoma, *IQR *Interquartile range

Table 2 displays the CT parameters of the patients who were enrolled in the study. The patients with aggressive pathology exhibited the following qualitative CT parameters: renal tumors located closer to the collecting system (*P* < 0.001), higher RENAL scores (*P* = 0.005), higher rates of CT necrosis (*P* < 0.001), calcification (*P* = 0.044), peritumoral neovascularity (*P* < 0.001), and irregular tumor margins (*P* < 0.001) in the image. We also evaluated quantitative CT data and determined that the ICC between the results measured by two reviewers was high, ranging from 0.758 to 0.982. Regarding TAV, TAC, and HDT, the ICC results were 0.824, 0.758, and 0.811 in PCP; 0.976, 0.982, and 0.895 in CMP; 0.951, 0.962, and 0.916 in NP; and 0.922, 0.963, and 0.914 in EP. Among all quantitative CT parameters, only TAV-CMP (*P* = 0.062) and HDT-NP (*P* = 0.058) were not statistically different between the two groups.

**Table 2:** CT parameters of enrolled patients

Variables		Overall (*N* = 776)	Non-aggressive pathology (*N* = 526)	Aggressive pathology (*N* = 250)	P
Maximal tumor diameter, cm (%) (R score)					0.472
≤4		389.0 (50.1)	293.0 (55.7)	96.0 (38.4)	
>4-<7		387.0 (49.9)	233.0 (44.3)	154.0 (61.6)	
Exophytic/endophytic rate (%) (E score)					0.469
≥50%		386.0 (49.7)	259.0 (49.2)	127.0 (50.8)	
<50%		254.0 (32.7)	179.0 (34.0)	75.0 (30.0)	
Endophytic		136.0 (17.5)	88.0 (16.7)	48.0 (19.2)	
Distance to the collecting system, mm (%) (N score)					< 0.001
>7		475.0 (61.2)	351.0 (66.7)	124.0 (49.6)	
4–7		79.0 (10.2)	57.0 (10.8)	22.0 (8.8)	
≤4		222.0 (28.6)	118.0 (22.4)	104.0 (41.6)	
Polar location (%) (L score)					0.056
Entirely above or below the polar line		226.0 (29.1)	164.0 (31.2)	62.0 (24.8)	
Cross the polar line		297.0 (38.3)	204.0 (38.8)	93.0 (37.2)	
>50% crosses the polar line crosses the axial renal midline or entirely between the polar lines		253.0 (32.6)	158.0 (30.0)	95.0 (38.0)	
RENAL score (%)					0.005
Low (4–6)		381.0 (49.1)	281.0 (53.4)	100.0 (40.0)	
Intermediate (7–9)		319.0 (41.1)	210.0 (39.9)	109.0 (43.6)	
High (10–12)		76.0 (9.8)	35.0 (6.7)	41.0 (16.4)	
CT necrosis (%)		306(39.4)	136(25.9)	170(68.0)	< 0.001
Calcification (%)		46.0 (5.9)	25.0 (4.8)	21.0 (8.4)	0.044
Tumor margin regularity (%)		169.0 (21.8)	48.0 (9.1)	121.0 (48.4)	< 0.001
Peritumoral neovascularity (%)		73.0 (9.4)	19.0 (3.6)	54.0 (21.6)	< 0.001
Pre-contrast phase (IQR)					
TAV-PCP		30.5 (26.0–35.0)	30.0 (26.0–35.0)	31.0 (28.0–35.0)	0.004
HDT-PCP		11.0 (9.0–14.0)	11.0 (9.0–14.0)	12.0 (9.0–14.0)	0.03
Corticomedullary phase (IQR)					
TAV-CMP		116.0 (86.0-151.8)	119.0 (88.0-156.0)	110.5 (84.8-148.3)	0.062
TEV-CMP		84.0 (57.0-121.0)	87.0 (60.0-123.3)	78.5 (54.0-78.5)	0.024
RER-CMP		0.7 (0.4–0.9)	0.7 (0.5-1.0)	0.6 (0.4–0.8)	< 0.001
HDT-CMP		40.0 (32.0–50.0)	39.0 (32.0–50.0)	42.0 (32.0–50.0)	0.041
Nephrographic phase (IQR)					
TAV-NP		101.0 (82.0-120.0)	104.0 (84.0-125.0)	97.0 (80.0-114.0)	< 0.001
TEV-NP		70.5 (52.0–90.0)	73.0 (55.0–93.0)	65.0 (48.0–83.0)	< 0.001
RER-NP		0.6 (0.4–0.7)	0.6 (0.5–0.7)	0.5 (0.4–0.6)	< 0.001
HDT-NP		27.0 (22.0–34.0)	27.0 (22.0–34.0)	29.0 (21.0–34.0)	0.058
Excretory phase (IQR)					
TAV-EP		81.0 (69.0–95.0)	82.0 (69.0–96.0)	80.0 (68.8–92.3)	0.043
TEV-EP		50.0 (38.0–63.0)	51.0 (39.0–65.0)	49.0 (36.8–61.0)	0.002
RER-EP		0.5 (0.4–0.6)	0.5 (0.4–0.6)	0.4 (0.3–0.5)	< 0.001
HDT-EP		20.0 (16.0–24.0)	19.0 (16.0–24.0)	21.0 (16.0–25.0)	0.006

*TAV *Attenuation value of renal tumor, *TEV *Net enhancement value of renal tumor, *RER *Relative enhancement ratio, *HDT *Heterogeneous degree of tumor, *IQR *Interquartile range

### Determination of independent predictors

As illustrated in Table 3, we included all clinicopathological and radiologic variables and completed the univariate and multivariate logistic regression analyses. The results of univariate analyses showed that age, ECOG performance status, clinical tumor size, hemoglobin, NLR, type of nephrectomy, distance to the collecting system, CT necrosis, tumor margin regularity, peritumoral neovascularity, TAV-PCP, TEV-CMP, RER-CMP, TAV-NP, TEV-NP, RER-NP, TEV-EP, RER-EP and HDT-EP were risk factors of aggressiveness for cT1 solid RCC (all *P* < 0.05). Then, we performed a collinearity test for the 19 variables selected from the univariate logistic regression and excluded 4 variables with VIF > 5 (TAV-NP, TEV-NP, TEV-EP, HDT-EP). After incorporating the 15 variables into the multivariate analysis, the result showed that NLR (*P* < 0.001), distance to the collecting system (*P* = 0.036), CT necrosis (*P* < 0.001), tumor margin irregularity (*P* < 0.001), peritumoral neovascularity (*P* = 0.001) and RER-NP (*P* = 0.047) were independent predictors of aggressive pathology for cT1 solid RCC.

**Table 3:** Univariate and multivariate logistic regression analyses of aggressive pathology for cT1 solid RCC.

Variables	Univariate			Multivariate		
	OR	95%CI	*P* value^a^	OR	95%CI	*P* value
**Clinical findings**						
Age at surgery, year	**1.025**	**1.011–1.040**	**< 0.001**			
Gender: male	1.058	0.755–1.482	0.765			
Laterality: left	0.910	0.673–1.230	0.571			
ECOG performance status: 2–4	**3.061**	**1.340–6.992**	**0.016**			
Symptomatic presentation	1.370	0.996–1.884	0.083			
Hypertension	1.016	0.752–1.373	0.917			
Diabetes	1.235	0.864–1.765	0.298			
Smoking history	1.179	0.867–1.605	0.341			
BMI, kg/m^2^	0.971	0.929–1.015	0.244			
Clinical tumor size, cm	**1.424**	**1.269–1.597**	**< 0.001**			
Hemoglobin, g/L	**0.983**	**0.974–0.991**	**< 0.001**			
NLR	**1.081**	**1.042–1.121**	**< 0.001**	**1.096**	**1.050–1.145**	**< 0.001**
**Surgical findings**						
Surgical approach			0.257			
Open	1(Reference)					
Laparoscopic	3.698	0.838–16.318	0.084			
Robotic	3.323	0.721–15.317	0.124			
Type of nephrectomy: Radical nephrectomy	**3.156**	**2.252–4.424**	**< 0.001**			
**Imaging findings**						
Maximal tumor diameter, cm			0.515			
≤4	1(Reference)					
>4-<7	0.895	0.662–1.210				
Exophytic/endophytic rate			0.529			
≥50%	1(Reference)					
<50%	0.854	0.606–1.204	0.369			
Endophytic	1.112	0.738–1.678	0.611			
Distance to the collecting system, mm			**< 0.001**			**0.036**
>7	**1(Reference)**			**1(Reference)**		
4–7	**1.093**	**0.641–1.861**	**0.745**	**0.548**	**0.280–1.072**	**0.079**
≤4	**2.495**	**1.787–3.483**	**< 0.001**	**1.360**	**0.888–2.083**	**0.158**
Polar location			0.086			
Entirely above or below the polar line	1(Reference)					
Cross the polar line	1.206	0.823–1.766	0.336			
>50% crosses the polar line, crosses the axial renal midline or entirely between the polar lines	1.590	1.080–2.343	0.019			
CT necrosis	**6.094**	**4.382–8.474**	**< 0.001**	**6.005**	**4.017–8.977**	**< 0.001**
Calcification	1.838	1.008–3.351	0.077			
Tumor margin regularity: irregular	**9.341**	**6.345–13.752**	**< 0.001**	**8.037**	**5.098–12.669**	**< 0.001**
Peritumoral neovascularity	**7.352**	**4.249–12.719**	**< 0.001**	**3.064**	**1.598–5.875**	**0.001**
Pre-contrast phase						
TAV-PCP	**1.030**	**1.008–1.052**	**0.016**			
HDT-PCP	1.045	1.004–1.087	0.056			
Corticomedullary phase						
TAV-CMP	0.997	0.994-1.000	0.083			
TEV-CMP	**0.994**	**0.994-1.000**	**0.047**			
RER-CMP	**0.394**	**0.259-0.600**	**< 0.001**			
HDT-CMP	1.011	1.000-1.022	0.072			
Nephrographic phase						
TAV-NP	0.991	0.986–0.996	0.003			
TEV-NP	0.989	0.983–0.994	< 0.001			
RER-NP	**0.112**	**0.048–0.261**	**< 0.001**	**0.133**	**0.047–0.374**	**0.047**
HDT-NP	1.004	0.989–1.020	0.084			
Excretory phase						
TAV-EP	0.993	0.985-1.000	0.080			
TEV-EP	0.989	0.981–0.996	0.010			
RER-EP	**0.115**	**0.045–0.298**	**< 0.001**			
HDT-EP	1.032	1.009–1.055	0.015			

*BMI *Body mass index, *NLR *Neutrophil-to-lymphocyte ratio, *RCC *Renal cell carcinoma, *TAV *Attenuation value of renal tumor, *TEV *Net enhancement value of renal tumor, *RER *Relative enhancement ratio, *HDT *Heterogeneous degree of tumor. ^a^All P values in univariate logistic regression are adjusted by Benjamini-Hochberg method.

### Construction and evaluation of the nomogram model

**Fig. 4:**
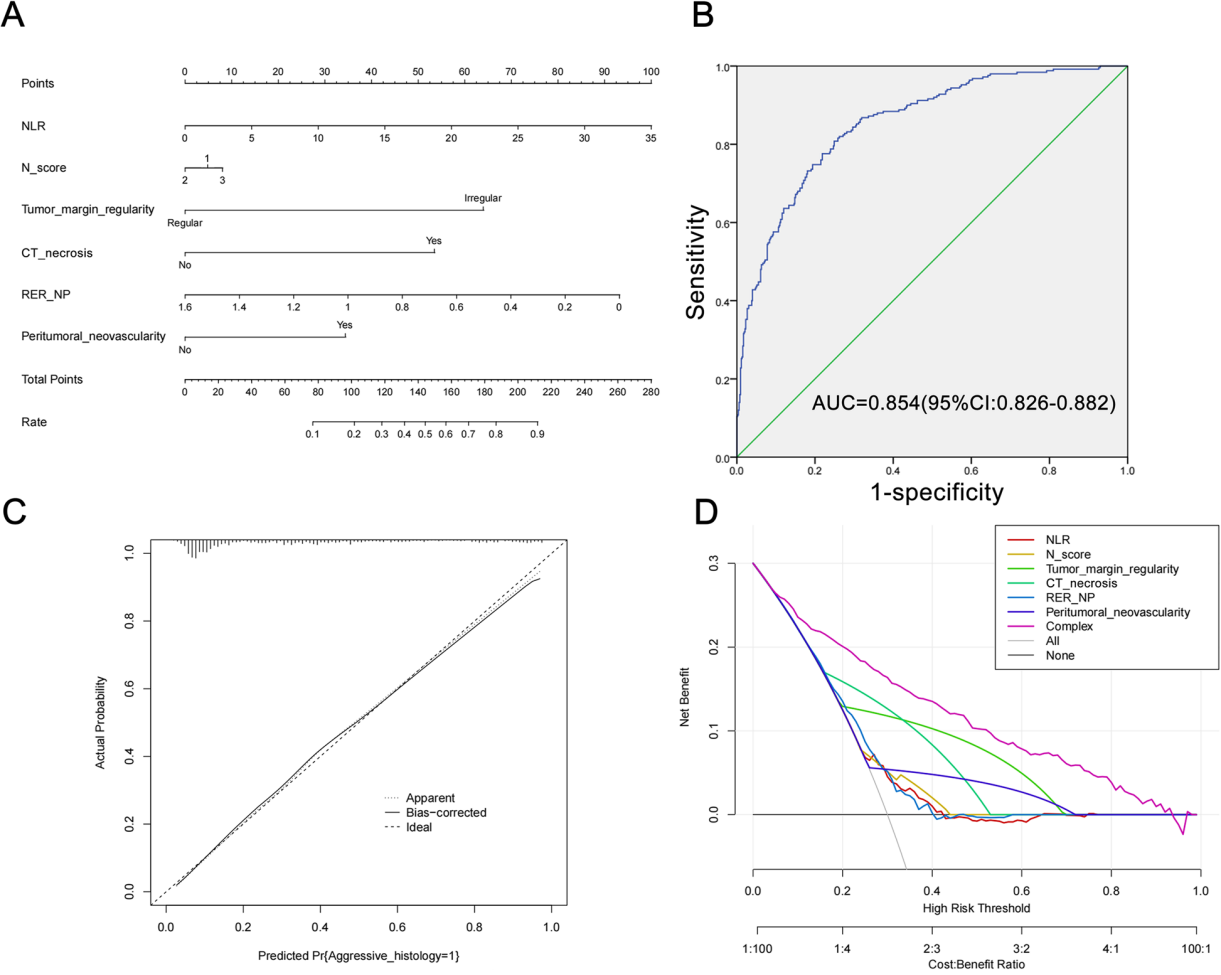
**A** Nomogram, (**B**) ROC curve, (**C**) calibration plot, and (**D**) DCA curve of the model for predicting aggressive pathology of cT1 solid RCC.  RCC: renal cell carcinoma; ROC: receiver operating characteristic; DCA: decision curve analysis.  A straight line was plotted from the corresponding location on each predictor to the “Points” to determine the points of a single predictor. The points of each predictor were summed to obtain a total point, then a straight line was plotted from the “Total Point” to the “Rate” to obtain the probability of aggressive pathology

After identifying the independent predictors of aggressiveness for cT1 solid RCC, we constructed a nomogram model, shown in Fig. 4A. The nomogram model had an AUC of 0.854 (95%CI: 0.826–0.882), with sensitivity and specificity of 0.808 and 0.751, respectively, indicating high discrimination of the model (Fig. 4B). The calibration plot of the nomogram model presented in Fig. 4C showed high consistency between the predicted and actual probability of aggressiveness for cT1 solid RCC. The Hosmer-Lemeshow test result was 0.645, revealing a good fit of the nomogram model. The DCA curve in Fig. 4D indicated that the net benefit of the nomogram model was significantly higher than that of a single variable. Then, we calculated the risk score according to the result of multivariate logistic regression. Risk score = 0.092×NLR + 0 (“N score”=1) /-0.602 (“N score”=2)/+0.307 (“N score”=3) + 0(without CT necrosis)/1.793 (with CT necrosis) + 0 (tumor margin regularity)/2.084 (tumor margin irregularity) + 0 (without peritumoral neovascularity) /1.120 (with peritumoral neovascularity) -2.020×RER_NP -1.559. We also calculated the predicted probability of aggressive pathology for cT1 solid RCC patients according to the formula between risk score and linear predictive probability: ln^(P/1−P)^ = risk score. Finally, the individual probability of aggressive pathology is calculated as follows: P (aggressive pathology) = 1/(1 + exp^−risk score^). We divided all cT1 solid RCC patients into the low-risk (*N* = 258), medium-risk (*N* = 259), and high-risk groups (*N* = 259) on average according to the P value, with the probability of aggressive pathology being 5.8% (15/258), 24.3% (63/259), and 66.4% (172/259), respectively. The cut-off P value was 0.105 between the low-risk and medium-risk groups and 0.400 between the medium-risk and high-risk groups. Subsequently, we performed subgroup analyses of cT1a and cT1b solid RCC patients according to the cut-off values of 0.105 and 0.400. The results showed that the probability of aggressive pathology was 6.2% (9/142), 26.9% (37/120), and 66.1% (84/127) for patients in the low-risk, medium-risk, and high-risk groups in the cT1a subgroup, and 5.3% (6/114),18.4% (26/141), and 66.7% (88/132) in the cT1b subgroup, respectively.

## Discussion

With the advancement of medical technology, there are now various treatment options available for T1 RCC patients, such as nephrectomy, active surveillance, RMB, radiofrequency ablation, cryoablation, and more. It is essential to provide personalized treatment plans for individuals with T1 RCC, taking into account their age, physical condition, and the anatomical and biological characteristics of their renal tumors. Research has shown that aggressive pathology of RCC is associated with a poor prognosis [3, 4, 21, 22]. Diagnostic imaging has advantages in distinguishing pathological aggressiveness, and preoperative CT enhancement and texture features are crucial for clinical decision-making in RCC cases. Because of the high accuracy of the Bosniak grading system in stratifying cystic renal masses as to the probability of malignancy, our study developed a nomogram model to predict postoperative pathology in cT1 solid RCC patients.

In our study, we contained 776 patients, and 32.2% of them had aggressive pathology, which is in line with previous findings ranging from 21.1–49% [15, 23]. We found that RCC patients with aggressive pathology were older, and had poorer ECOG performance status, higher NLR, higher rate of RN, higher clinical tumor size, and lower hemoglobin. The predictive value of tumor size has been confirmed in earlier reports, Ball et al. found tumor size to be an important risk factor for aggressive histology in cT1a RCC, and Bhindi et al. also reported tumor size-based risk stratification of the probability of adverse pathology in renal masses [8, 24]. Aggressive pathology was more common in older patients and resulted in increased frailty and comorbidities [16, 25]. In our study cohort, RCC patients with aggressive pathology had more complex tumor anatomy and consequently received a higher proportion of RN to reduce the likelihood of postoperative recurrence. The relationship between systemic inflammation markers and RCC pathology has been previously investigated, and hemoglobin and NLR have been confirmed as independent predictors of unfavorable pathology in cT1 RCC [23]. As the only clinical predictor included in our nomogram model, NLR is also a well-known prognostic biomarker in various solid tumors, such as RCC, lung cancer, gastric cancer, etc. [26, 27]. 

In a recent study, Ficarra et al. summarized several qualitative CT features with a significant role in predicting aggressive pathology of RCC. They found that clinical tumor size, tumor growth rate, enhancement characteristics, tumor margins, CT necrosis, and distance to the renal sinus are relevant features in predicting the biological aggressiveness of RCC, and that peritumoral and intratumoral neovascularity are variables that need to be further accessed [18]. Our data confirmed that tumor margin irregularity, CT necrosis, distance to the collecting system, and peritumoral neovascularity were independent predictors of aggressive pathology for cT1 solid RCC. However, tumor growth rate could not be recorded in our study since all patients underwent nephrectomy without active surveillance. Although RCC patients with aggressive pathology had a significantly larger clinical tumor size, it was excluded from the final nomogram model after multivariate regression. This may be because our patients with cT1 RCC have other predictors that are more strongly associated with aggressiveness, as the relationship between tumor size and aggressiveness was stronger in T2 and advanced RCC. Radiologists recommend that renal tumor margins should be classified into “circumscribed” and “irregular” [28]. In our study, tumor margin irregularity includes three categories nodular growth pattern, blurred boundary between renal tumor and parenchyma, and completely non-elliptical shape, which are summarised by previous literature [10, 16, 29]. The presence of irregular renal tumor margins has been shown to be a strong predictor of perirenal/renal sinus fat invasion, pT3a upstage, and aggressive histology subtype [10, 29, 30]. Our findings align with previous literature and confirm that irregular renal tumor margin is an important independent risk factor of aggressive pathology for cT1 solid RCC. Moreover, as an important part of the RENAL nephrometry score, the distance of the renal tumor to the collecting system less than 4 mm in the image implies a higher rate of renal sinus fat invasion and upstaging to pT3a. Its potential role in predicting high nuclear grade and aggressive histological subtypes of RCC has also been reported and confirmed [30, 31]. Interestingly, our results suggest that compared to the distance greater than 7 mm from the collecting system, cT1 RCC with a distance between 4 and 7 mm has a lower probability of aggressive pathology, due to its lower rate of perirenal fat invasion. Previous studies have demonstrated that CT necrosis of renal masses was closely related to pT3a RCC, high nuclear grade, sarcomatoid dedifferentiation, and aggressive histological subtypes [30, 32–34]. Similarly, our study arrived at comparable outcomes. Peritumoral neovascularity was not routinely reported by radiologists, but it may have potential predictive value. Recently, Yanagi et al. reported that peritumoral neovascularity was a significant factor associated with tumor recurrence in patients with small renal masses, and Suo et al. found that RCC patients with peritumoral neovascularity had higher pT stage, nuclear grade, and shorter OS [20, 35]. There is increasing evidence that the high level of peritumoral angiogenesis is associated with aggressiveness of RCC and our study confirmed that peritumoral neovascularity could be interpreted as a predictor of aggressiveness [36].

To our acknowledgment, several studies have utilized quantitative CT-derived parameters to differentiate histological subtypes, nuclear grade, and prognosis [37–39]. However, this is the first study to combine qualitative and quantitative CT parameters for the prediction of aggressive pathology in RCC. Coy et al. reported that clear cell RCC with high nuclear grade had lower enhancement values in NP and EP, and absolute enhancement < 52 HU was an independent predictor of high nuclear grade in NP [39]. Similarly, Zhu et al. identified age, irregular tumor margin, and low tumor enhancement as independent predictors of high tumor grade [37]. We also found statistically significant lower TAV, TEV, and RER of aggressive RCC in NP and EP, which may be due to the difference of microvessel density and micronecrotic areas between aggressive and non-aggressive RCC [37, 39, 40]. Because of the less enhancement of renal tumor compared to the surrounding renal cortex, the NP was commonly considered as the most sensitive phase for characterizing renal masses. We incorporated renal cortex enhancement as the reference and found that RER-NP was negatively correlated with aggressive pathology as an independent predictor in cT1 solid RCC. Furthermore, we found that HDT of RCC was higher in patients with aggressive pathology. The relatively high local necrosis and ischemic change of RCC with aggressiveness result in the decrease of attenuation value in this area, which exhibits enhanced heterogeneity in the ROI region. Additionally, the aggressive pathological features make the blood supply within RCC unbalanced, and the distribution degree of iodine agent within the tumor varies largely, leading to a significantly higher HDT value. Based on the previous literature review, we included as many variables as possible that may be relevant to aggressive pathology of RCC. No variables related to the EP were included in the final nomogram. Given that not all medical centers include the EP in contrast-enhanced CT when evaluating renal masses that do not invade the collecting system, our nomogram can be applied without limitations in these medical centers. Ultimately, our nomogram model had an AUC of 0.854, diagnostic sensitivity and specificity of 0.808 and 0.751, with good calibration and net clinical benefit. Our model outperforms other existing predictive methods for RCC aggressive pathology [8, 23, 24, 30]. The individual predictive probability of aggressive pathology, based on our nomogram model, was also calculated and proved to be significant for guiding clinical treatment. Utilizing 0.105 and 0.400 as the cut-off values for the low-risk, medium-risk, and high-risk groups demonstrated excellent risk stratification capability in cT1 solid RCC patients, as well as within the cT1a and cT1b subgroups. Our results recommend active surveillance for cT1 solid RCC patients with linear predictive probability values less than 0.105, and PN/RN for patients with linear predictive probability values greater than 0.400.

There are certain limitations to our research. Firstly, our study was a retrospective analysis carried out in a single institution, which may cause selection bias. Our nomogram model still needs to be further validated with samples from other medical centers. Secondly, different patients have varying abilities to metabolize contrast agents, leading to individual differences in the enhanced scanning time, which may affect the quantitative CT parameters. Although we applied renal cortex CT values as the reference and correction, further research is needed to eliminate the impact of scanning time on the final diagnostic performance. Thirdly, our study only included cT1 RCC patients and focused on the impact of contrast-enhanced CT on pathology. In future studies, cT2 and locally advanced RCC patients should also be included. Other emerging imaging methods, such as radiomics texture analysis and 3D reconstruction, should also be applied and compared to explore the differences and correlations among these methods in imaging-based diagnosis.

In conclusion, based on CT features and clinical data, we have developed a nomogram model that can predict the risk of aggressive pathology in cT1 solid RCC patients accurately. Our nomogram model could be used to calculate the individualized risk of aggressive pathology and provide treatment decisions for cT1 solid RCC patients.

## Data Availability

The datasets used and/or analyzed during the current study are available from the corresponding author on reasonable request.

## Abbreviations

RCC: Renal cell carcinoma
PN: Partial nephrectomy
RN: Radical nephrectomy
ROI: Region of interest
ROC: Receiver operating characteristic
DCA: Decision curve analysis
ICC: Interclass correlation coefficient
NLR: Neutrophil-to-lymphocyte ratio
AUC: Area under the curve
CT: Computed tomography
OS: Overall survival
CSS: Cancer-specific survival
RMB: Renal mass biopsy
PCP: Pre-contrast phase
CMP: Corticomedullary phase
NP: Nephrographic phase
EP: Excretory phase
TAV: Attenuation value of renal tumor
TAC: Attenuation value of renal cortex
TEV: Net enhancement value of renal tumor
CEV: Net enhancement value of renal cortex
RER: Enhancement ratio
HDT: Heterogeneous degree of tumor
BMI: Body mass index
SD: Standard deviation
IQR: Interquartile range
